# Comparative analysis of intestinal bacteria among venom secretion and non-secrection snakes

**DOI:** 10.1038/s41598-019-42787-6

**Published:** 2019-04-19

**Authors:** Zuodong Qin, Siqi Wang, Dezhi Guo, Jialiang Zhu, Huahai Chen, Le Bai, Xiaofang Luo, Yeshi Yin

**Affiliations:** 1 Key Laboratory of Comprehensive Utilization of Advantage Plants Resources in Hunan South, College of Chemistry and Bioengineering, Hunan University of Science and Engineering, Yongzhou, Hunan China; 2 Yongzhou City Strange Snake Science and Technology Industrial Co., Ltd., Yongzhou, Hunan 425000 China

**Keywords:** Environmental microbiology, Microbial ecology

## Abstract

To further investigate the bacterial community and identify the bacterial biomarkers between venom secretion and non-venom secretion snakes, 50 intestinal samples (25 large intestine, 25 small intestine) were obtained from 29 snakes (13 gut samples from *Deinagkistrodon*, 26 from *Naja* and 11 from *Ptyas mucosa*). 16S rDNA high-throughput sequencing results showed that 29 bacterial phyla, 545 bacterial genera, and 1,725 OTUs (operational taxonomic units) were identified in these samples. OTU numbers and the Ace, Chao, Shannon, and Simpson indexes were very similar among the three breeds of snakes included in this study. The *Bacteroidetes*, *Firmicutes*, *Fusobacteria* and *Proteobacteria* were predominant bacterial phyla. The relative abundance at the phylum level among these samples was similar, and the difference between small and large intestinal samples was not obvious. However, at the genus level, venom secretion snakes *Deinagkistrodon* and *Naja* clustered together according to different breeds. 27, 24, and 16 genera were identified as core microbes for *Deinagkistrodon*, *Naja*, and *Ptyas mucosa*, respectively. Interestingly, the relative abundances of genera *Hafnia_Obesumbacterium*, *Providencia*, and *Ureaplasma* were found to be significantly higher in non-venom secretion snakes, and the genera *Achromobacter*, *Cetobacterium*, *Clostridium innocuum* group, *Fusobacterium*, *Lachnoclostridium*, *Parabacteroides*, and *Romboutsia* were only detected in venom secretion snakes. The function of these bacteria in venom secretion needs to be further studied, and these venom secretion related genera may be the promising target to improve venom production.

## Introduction

Gut microbiota refer to a large number of microorganisms existing in the intestine of human and animals. Gut microbitoa take part in host metabolization and regulate host immune systems development, which plays a very important role in maintaining the host health balance^1,2^. Many diseases have been reported to be associated with changes in diversity or richness of gut microbiota^3^. So far, most of the studies have focused on mammalian vertebrates’ gut microbiota, with fewer investigations on reptile vertebrates such as snake. As one of the special economic animals, snake has high commercial values as its whole body has value. Snake gall is regarded as a precious Chinese medicine, which can be used to treat damp and fire, improve eyesight, relieve a cough, and reduce phlegm^4^. Antithrombotic enzyme obtained from *Agkistrodon* can be utilized to treat thrombus^5,6^. In particular, known as “liquid gold” in the international market, and costing dozens of times more than gold, snake venom is insufficiently available as animal medicine in the international market. Due to its proven antivirus^7^, anti-inflammatory^8^, and immune regulatory^8^ functions, snake venom not only has been in use as tranquilizer^9^, and for colorectal cancer treatment^10^, but also can be developed into health care products such as snake wines^11,12^.

Although diet elements or chemical compounds can be metabolized by microbes or co-metabolized by the host and microbes^13,14^, the relationship between venom secretion and gut microbiota requires further study. To investigate the flora difference between venom-secretion and non-venom secretion snakes, the gut samples of three kinds of snakes (e.g., *Deinagkistrodon*, *Naja*, and *Ptyas mucosa*) were collected for high-throughput 16 s rDNA sequencing analysis in this study. Our results revealed *Bacteroidetes*, *Firmicutes*, *Fusobacteria*, *Proteobacteria*, *Actinobacteria*, and *Saccharibacteria* to be the major bacteria present in these three snakes. Some core microbes, which can be detected in all samples, were identified in this study. Some bacteria may be related to the venom secretion by comparison analysis between two venom secretion snakes (*Deinagkistrodon* and *Naja*) and a non-venom secretion snake (*Ptyas mucosa*).

## Materials and Methods

### Sample collection

A total of five small intestinal samples and six large intestinal samples were collected from seven *Ptyas mucosa*; seven small intestinal samples and six large intestinal samples were collected from eight *Deinagkistrodon*; 13 small intestinal samples and 13 large intestinal samples were collected from 14 *Naja*. All of these samples were used for bacterial genome DNA extraction and high-throughput 16S rDNA sequencing. Detailed information of these snakes, like gender, weight and length *et al*., is listed in Supplementary Table 1. As in routine procedures, snakes were anesthetized using diethyl ether and then were killed by severing the spinal cord immediately posterior to the head. After the snakes were sacrificed, the snake skin was cleaned and wiped with sterile alcohol, and the gastrointestinal tract of these snakes was exposed via a mid-abdomen incision. The contents of the small intestine and large intestine were drained into different sterile vials. The collected samples were quickly frozen in liquid nitrogen, and then stored at −80 °C for further analysis. The study was approved by the Ethics Committee of the Hunan University of Science and Engineering, and all procedures were performed according to the relevant guidelines and regulations.

### Bacterial genomic DNA extraction

Bacterial genomic DNA was extracted using a QIAamp DNA Stool Mini Kit following the manufacturer’s instructions (QIAGEN, Germany). The concentration of the extracted DNA was determined by using a NanoDrop ND-2000 (NanoDrop Technologies, USA), and its integrity and size were confirmed by performing 1% agar gel electrophoresis.

### 16S rRNA gene high-throughput sequencing and analysis

According to previous work, bacterial genome DNA samples extracted from snake gut samples were amplified using barcoded 16S rRNA gene primers 338F (5′-ACT CCT ACG GGA GGC AGC A-3′) with 806R (5′-GGA CTA CHV GGG TWT CTA AT-3′)^15^. After adding sequence adapts and constructing sequencing libraries, next-generation sequencing was performed on an Illumina MiSeq 300PE system that was operated by XY Biotechnology Co., Ltd., Shanghai, China. In total, 50 snake gut samples were sent to high-throughput 16S rRNA gene sequencing.

The original image data from the second-generation high-throughput sequencing instruments were converted into sequence data by base calling and saved as in a FASTQ format. Next-generation sequencing reads were identified by barcodes using a QIIME pipeline. Clean and high-quality sequences were then used for downstream analysis. A 97% similarity cutoff was employed in defining OTUs using Mothur^16^. One sequence was picked out from each OTU as representative. The representative sequences were classified using the RDP classifier method^17,18^ and SILVA database (https://www.arb-silva.de/documentation/release-123/). Good’s coverage, alpha diversities including Simpson and Shannon index, and richness (observed number of OTUs) values were calculated using Mothur. The data have been deposited in the sequence read archive (SRA) of NCBI as GenBank Accession Number SRP132421. Basic data on 16S rRNA gene high-throughput sequencing are listed in Supplementary Table 2.

### Statistical Analysis

SPSS software (version 20.0; SPSS Inc., USA) and the ANOVA LSD(L) test were employed in this study. *P* < 0.05 was considered to be statistically significant. Plot cladograms and significantly different bacterial taxa were analyzed using a LDA Effect Size (LEfSe) algorithm^19^.

## Results

### Barcode 16s pyrosequencing and diversity analysis

**Figure 1:**
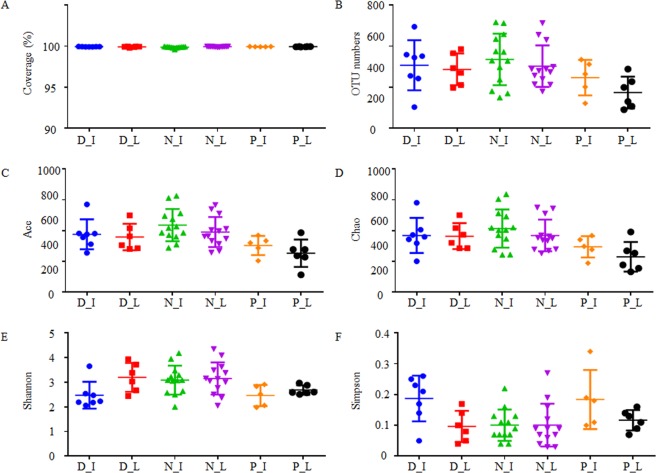
Alpha diversity analysis of sequencing samples. D, N, and P refer to *Deinagkistrodon*, *Naja*, and *Ptyas mucosa*, respectively. I and L represent ileum and large intestinal samples, respectively.

In this study, 50 gut samples collected from three snake species, *Deinagkistrodon*, *Naja*, and *Ptyas mucosa*, were subjected to the 16s rDNA high-throughput sequencing analysis. In total, 1,301,357 valid reads were obtained from 13 *Deinagkistrodon* gut samples, 2,461,474 valid reads were obtained from 26 *Naja* gut samples, and 971,049 valid reads were obtained from 11 *Ptyas mucosa* gut samples (Supplementary Table 2). Of these sequences, 94.4%, 94.6%, and 99.9% reads were classified as bacterial phyla for *Deinagkistrodon*, *Naja*, and *Ptyas mucosa* samples, respectively. The reads that could be classified as bacteria were used for further analysis. As shown in Fig. 1A and Supplementary Figure 1, the sequencing coverage was close to 100% and the rarefaction curves exhibits a tendency to saturation, indicating that most bacterial species were captured by sequencing in all samples.

After classification, 29 bacterial phyla, 545 bacterial genera, and 1,725 OTUs were identified from these snake gut samples. In this study, 97% similarity was set as the OTU cut-off point. For each kind of snakes, 104–495 OTUs, 150–517 OTUs, and 91–335 OTUs were identified in *Deinagkistrodon*, *Naja*, and *Ptyas mucosa* samples, respectively (Supplementary Table 2). The distribution of OTU numbers in each sample is shown in Fig. 1B. The number of OTUs was slightly higher in venom secretion snakes *Deinagkistrodon* and *Naja* than non-venom secretion snake *Ptyas mucosa*. Although the difference of small intestinal samples was not statistically significant among these groups, OTU numbers were greatly higher in *Naja* than in *Ptyas mucosa* for the large intestinal samples (Table 1).

**Table 1:** Statistic analysis of OTU numbers and alpha diversity indexes*.

	D_I:N_I	D_I:P_I	N_I:P_I	D_L:N_L	D_L:P_L	N_L:P_L
OTU index	NS	NS	NS	NS	NS	0.01
Ace index	NS	NS	0.02	NS	NS	NS
Chao index	NS	NS	0.02	NS	0.03	0.01
Shannon index	0.03	NS	0.04	NS	NS	NS
Simpson index	0.01	NS	NS	NS	NS	NS

*D, N, and P refer to *Deinagkistrodon*, *Naja*, and *Ptyas mucosa*, respectively. I and L represent ileum and large intestinal samples, respectively. Numbers in this table are the p value for two tailed hypothesis testing; NS means the difference between samples was not significant.

To analyze the alpha diversity of these samples, the indexes of Ace, Chao, Shannon, and Simpson were calculated. This process allowed us to fully characterize the bacterial community diversity in samples. Detailed information on the estimators for each sample is presented in Supplementary Table 2. As shown in Fig. 1C–F, while the indexes of Ace, Chao, and Shannon were slightly higher in *Deinagkistrodon* and *Naja* samples than *Ptyas mucosa*, the index of Simpson appears to be higher in *Ptyas mucosa* samples. Statistical analysis results showed that Ace, Chao, and Shannon indexes were significantly different between *Naja* and *Ptyas mucosa* small intestinal samples; Shannon and Simpson were significantly different between *Deinagkistrodon* and *Naja* small intestinal samples; Chao index was significantly different both between *Deinagkistrodon* to *Ptyas mucosa, and Deinagkistrodon* to *Naja* large intestinal samples (Table 1).

**Figure 2:**
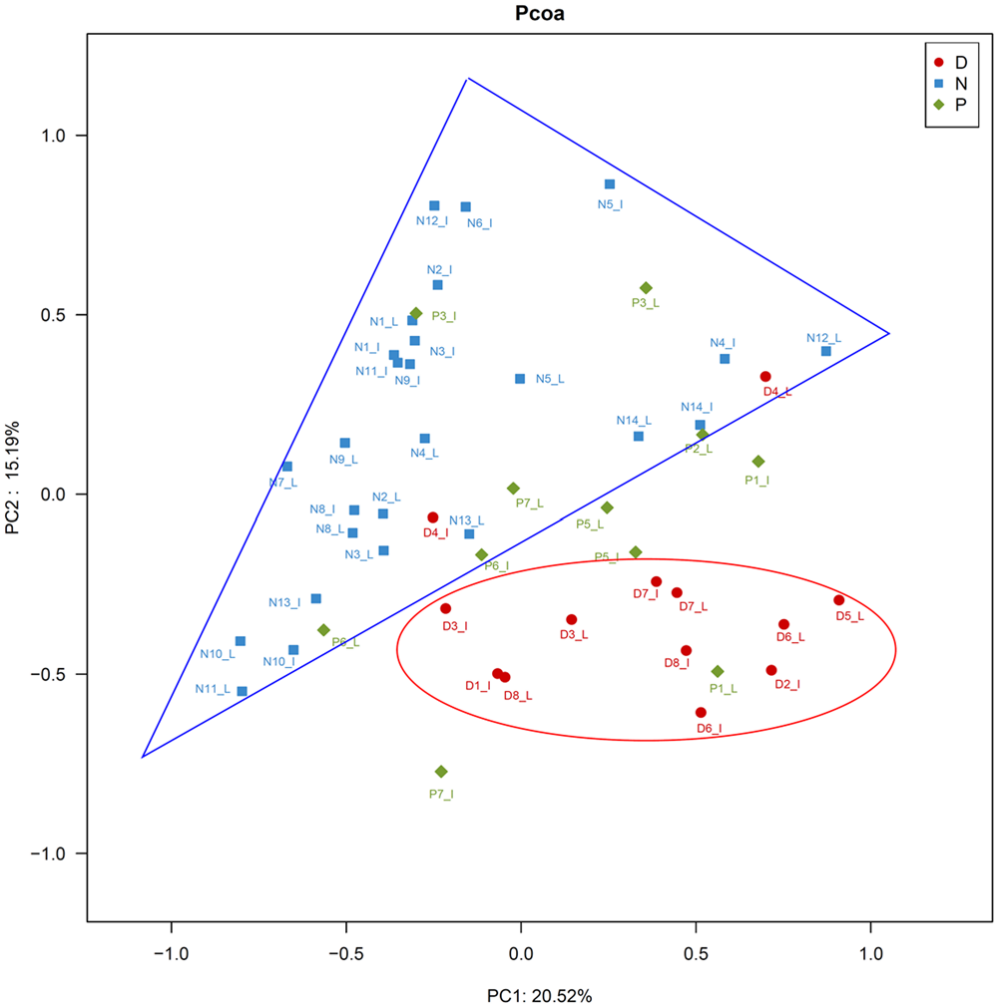
Principal coordinates analysis (PCoA) plot. PCoA plot of the gut microbiota based on the unweighted UniFrac metric. D, N, and P refer to *Deinagkistrodon*, *Naja*, and *Ptyas mucosa*, respectively. I and L represent ileum and large intestinal samples, respectively.

To analyze the beta diversity of the snake gut samples in this study, principal coordinate analysis (PCoA) was employed. As illustrated in Fig. 2, PCoA results indicated that most of the samples from *Deinagkistrodon* and *Naja* groups were clustered together with those of *Ptyas mucosa* groups being more dispersive.

### Microbial composition and core gut microbes of snake gut samples

**Figure 3:**
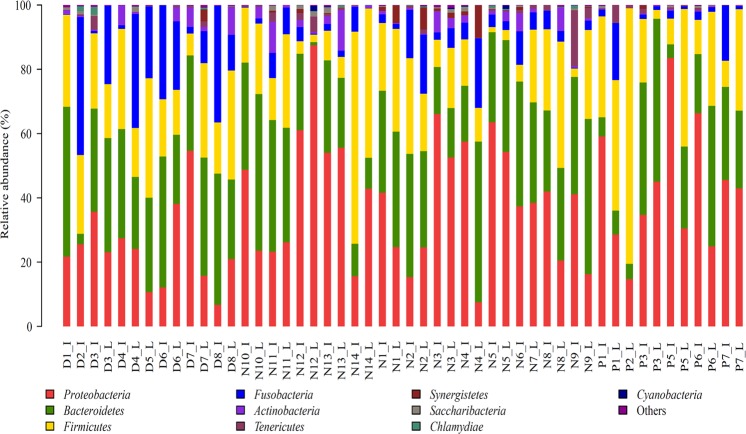
Distribution of bacterial phyla among 50 sequenced snake gut samples. The top 10 phyla in all sequenced samples are presented. D, N, and P refer to *Deinagkistrodon*, *Naja*, and *Ptyas mucosa*, respectively. I and L represent ileum and large intestinal samples, respectively.

Ten bacterial phyla with a relative abundance higher than 1% were identified from sequence samples. Among them, six phyla *Bacteroidetes*, *Firmicutes*, *Fusobacteria*, *Proteobacteria*, *Actinobacteria*, and *Saccharibacteria* were detected in all samples. As shown in Fig. 3, the relative abundances of the top 10 bacterial phyla accounted for most of the sequenced reads. The phyla *Bacteroidetes*, *Firmicutes*, *Fusobacteria*, and *Proteobacteria* were the predominant bacteria, which accounted for 80.5–99.9% of the reads of all sequenced samples, with *Actinobacteria* and *Saccharibacteria* very low (Supplementary Figure 2). In 46 of 50 samples, more than 90% of sequenced reads were identified as bacterial phyla *Bacteroidetes*, *Firmicutes*, *Fusobacteria*, and *Proteobacteria*. The average percentages of these phyla in *Deinagkistrodon*, *Naja*, and *Ptyas mucosa* samples were 30.5%, 29.2%, and 23.2% for *Bacteroidetes*; 22.7%, 19.1%, and 27.7% for *Firmicutes*; 18.2%, 5.5%, and 4.4% for *Fusobacteria*; 24.4%, 40.1%, and 43.3% for *Proteobacteria*. Although the difference of the phyla *Bacteroidetes* and *Firmicutes* among *Deinagkistrodon*, *Naja*, and *Ptyas mucosa* samples was not significant, the relative abundance of phylum *Fusobacteria* was significantly higher in *Deinagkistrodon* than in *Naja* and *Ptyas mucosa* samples, and the relative abundance of the phylum *Proteobacteria* was significantly lower in *Deinagkistrodon* samples (Supplementary Figure 3A and B). Difference in some other minor bacterial phyla were also found from the pairwise comparison. *Synergistetes* was significantly higher in *Deinagkistrodon* than in *Naja* samples (Supplementary Figure 3A); *Actinobacteria* was significantly higher in *Deinagkistrodon* than in *Ptyas mucosa* samples (Supplementary Figure 3B); *Actinobacteria*, *Synergistetes* and *Chlamydiae* were significantly higher in *Naja* than in *Ptyas mucosa* samples (Supplementary Figure 3C).

**Figure 4:**
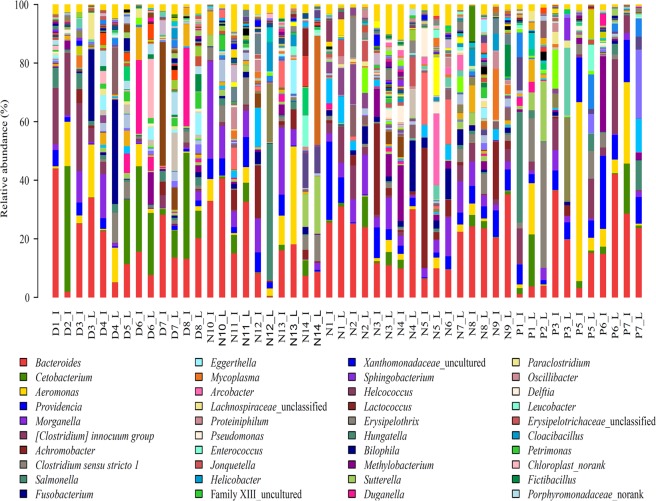
Bacterial community abundance at genus level. D, N, and P refer to *Deinagkistrodon*, *Naja*, and *Ptyas mucosa*, respectively. I and L represent ileum and large intestinal samples, respectively.

At the genus level, 108 genera with relative abundance higher than 1% were identified from sequence samples. The first top 10 genera, *Bacteroides*, *Cetobacterium*, *Aeromonas*, *Providencia*, *Morganella*, *Clostridium innocuum* group, *Achromobacter*, *Clostridium sensu stricto* 1, *Salmonella* and *Fusobacterium* (Fig. 4), accounted for 57.0%, 52.2% and 64.3% of sequenced reads in *Deinagkistrodon*, *Naja*, and *Ptyas mucosa* samples, respectively.

**Figure 5:**
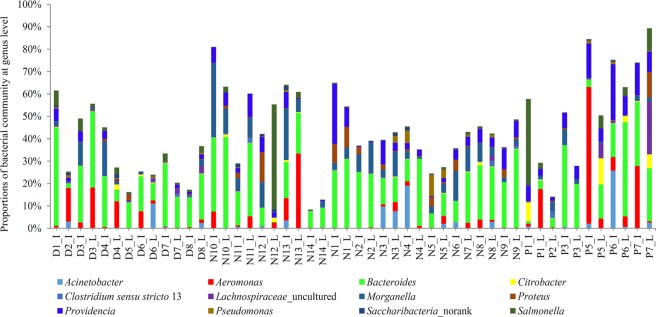
Distribution of core genera among 50 sequenced snake gut samples. Twelve genera shared by all sequenced samples are present. D, N, and P refer to *Deinagkistrodon*, *Naja*, and *Ptyas mucosa*, respectively. I and L represent ileum and large intestinal samples, respectively.

The genera that were detected in each sample were considered as core genera. As summarized in Table 2, 27, 24, and 16 genera were identified as core microbes for *Deinagkistrodon*, *Naja*, and *Ptyas mucosa*, respectively. The relative abundance of the core genera accounted for more than 50% of the sequence reads in most of the sequenced samples (Supplementary Figure 4A–C). 12 genera were detected in all sequenced snake samples, independent of breed (Table 2). The distribution of the 12 genera in all samples is presented in Fig. 5. The average abundance of the 12 core genera was upto 43.9%, and most of the genera like *Acinetobacter*^20^, *Aeromonas*^21^, *Citrobacter*^22^, *Morganella*^23^, *Proteus*^24^, *Providencia*^25^, *Pseudomonas*^26^, and *Salmonella*^27^ were human and animal pathogens or opportunistic pathogens (Table 2). Interestingly, genera *Achromobacter*, *Cetobacterium*, *Clostridium innocuum* group, *Fusobacterium*, *Lachnoclostridium*, *Parabacteroides*, *Romboutsia* can only be detected in venom secretion snakes *Deinagkistrodon* and *Naja* (Table 2).

**Table 2:** Core microbes detected in *Deinagkistrodon*, *Naja*, and *Ptyas mucosa* gut samples*.

*Deinagkistrodon* core genera	*Naja* core genera	*Ptyas mucosa* core genera
*Acinetobacter* (Y)	*Acinetobacter* (Y)	*Acinetobacter* (Y)
*Aeromonas* (Y)	*Aeromonas* (Y)	*Aeromonas* (Y)
*Bacteroides* (Y)	*Bacteroides* (Y)	*Bacteroides* (Y)
*Citrobacter* (Y)	*Citrobacter* (Y)	*Citrobacter* (Y)
*Clostridium sensu stricto 1*	*Clostridium sensu stricto 1*	*Clostridium sensu stricto 1*
*Lachnospiraceae_uncultured*	*Lachnospiraceae_uncultured*	*Lachnospiraceae_uncultured*
*Morganella* (Y)	*Morganella* (Y)	*Morganella* (Y)
*Proteus* (Y)	*Proteus* (Y)	*Proteus* (Y)
*Providencia* (Y)	*Providencia* (Y)	*Providencia* (Y)
*Pseudomonas* (Y)	*Pseudomonas* (Y)	*Pseudomonas* (Y)
*Saccharibacteria_norank*	*Saccharibacteria_norank*	*Saccharibacteria_norank*
*Salmonella* (Y)	*Salmonella* (Y)	*Salmonella* (Y)
*Achromobacter* (Y)	*Achromobacter* (Y)	*Edwardsiella* (Y)
*Cetobacterium*	*Cetobacterium*	*Erysipelothrix* (Y)
*Clostridium innocuum group* (Y)	*Clostridium innocuum group* (Y)	*Myroides* (Y)
*Fusobacterium* (Y)	*Fusobacterium* (Y)	*Ureaplasma* (Y)
*Lachnoclostridium*	*Lachnoclostridium*	
*Parabacteroides*	*Parabacteroides*	
*Romboutsia*	*Romboutsia*	
*Clostridium sensu stricto 13*	*Enterococcus* (Y)	
*Delftia*	*Eubacterium fissicatena group*	
*Epulopiscium*	*Hungatella*	
*Erysipelotrichaceae_unclassified*	*Shewanella*	
*Family XIII_uncultured*	*Xanthomonadaceae_uncultured*	
*Lachnospiraceae_unclassified*		
*Methylobacterium*		
*Porphyromonadaceae_uncultured*		

*Y in bracket represent that some species in this genus were opportunistic pathogens or related to infectious diseases. All of this kind of information was obtained by searching these bacteria on the website of https://en.wikipedia.org/wiki/Main_Page.

Although, some genera can be identified as core microbes, the difference among different breeds was obvious. Between *Deinagkistrodon* and *Naja*, 42 genera were significantly higher in *Deinagkistrodon* samples, and another 56 genera were significantly higher in *Naja* samples (Supplementary Figure 5A). Between *Deinagkistrodon* and *Ptyas mucosa*, 49 genera were significantly higher in *Deinagkistrodon* samples, and another 10 genera were significantly higher in *Ptyas mucosa* samples (Supplementary Figure 5B). Between *Naja* and *Ptyas mucosa*, 68 genera were significantly higher in *Naja* samples, and another 11 genera were significantly higher in *Ptyas mucosa* samples (Supplementary Figure 5C).

### Intestinal flora difference among studied snakes

**Figure 6:**
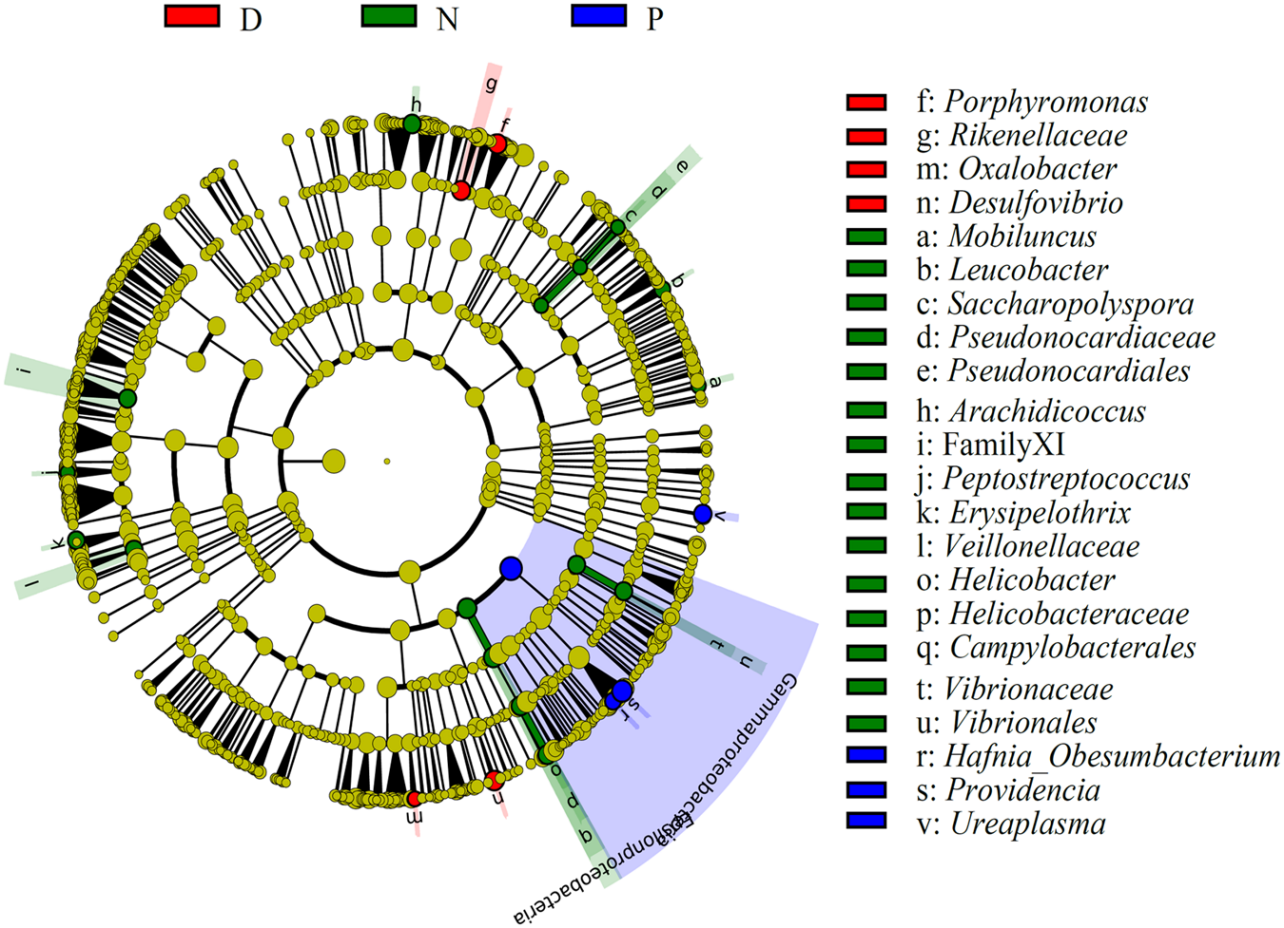
Analysis of different abundant bacterial taxa using LDA Effect Size (LEfSe) algorithm. The bacterial percentage of snake gut samples was used for LefSe analysis. The *P* value < 0.05 was identified as significantly different among these groups. Significantly enriched bacterial order, family and genus are listed on the right side. D, N, and P refer to *Deinagkistrodon*, *Naja*, and *Ptyas mucosa*, respectively.

Based on LEfSe analyses, the significantly different bacterial taxa (*P* < 0.05) among the three communities were identified and listed in the right side of the cladogram (Fig. 6). At the Class level, *Epsilonproteobacteria* was higher in *Naja* than other two breeds, and *Gammaproteobacteria* was higher in *Ptyas mucosa* than other two breeds. At the Order level, *Vibrionales*, *Campylobacterales*, and *Pseudonocardiales* were significantly higher in *Naja* samples. At the Family level, *Rikenellaceae* was significantly higher in *Deinagkistrodon* samples, with *Helicobacteraceae*, *Family XI* under Order *Clostridiales*, *Veillonellaceae*, and *Pseudonocardiaceae* significantly higher in *Naja* samples. At the genus level, uncultured bacteria under Family *Flavobacteriaceae*, *Oxalobacter*, other unidentified bacteria under Family *Lachnospiraceae*, *Porphyromonas* and *Desulfovibrio* were significantly higher in *Deinagkistrodon* samples; *Helicobacter*, *Leucobacter*, *Arachidicoccus*, *Peptostreptococcus*, *Erysipelothrix*, *Saccharopolyspora*, and *Mobiluncus* were significantly higher in *Naja* samples; *Providencia*, *Hafnia_Obesumbacterium*, and *Ureaplasma* were significantly higher in *Ptyas mucosa* samples (Fig. 6).

## Discussion

Although a few studies have used high-thoughput sequencing method to investigate the bacterial communities of *Agkistrodon piscivorus*^28^, *Crotalus horridus*^29^ and *Burmese pythons*^30^, no bacterial taxa data of gut microbiota from *Deinagkistrodon*, *Naja* and *Ptyas mucosa* have been reported so far. In this study, 29 bacterial phyla, 545 bacterial genera, and 1,725 OTUs were identified in the gut samples of these snakes. The average OTU number of these samples was about 300 to 400, which was similar to the OTU numbers detected in *Burmese pythons* samples^30^, but a little bit higher than *Agkistrodon piscivorus* samples^28^ for which about 100 OTUs were detected. The OTU number difference among our study with previously published reports maybe due to their different life habits, or dependence on the sequence depth.

In this study, the phyla *Bacteroidetes*, *Firmicutes*, *Fusobacteria*, and *Proteobacteria* were the predominant *bacteria*, which were consistent with results obtained from *Agkistrodon piscivorus*^28^ and *Crotalus horridus*^29^. *Bacteroidetes*, *Firmicutes*, and *Proteobacteria* were reported as the predominant bacteria detected in *Agkistrodon piscivorus* samples^28^, and *Proteobacteria* and *Firmicutes* were reported as the predominant bacteria detected in *Crotalus horridus* samples^29^. However, *Proteobacteria* was the major bacterial phylum, accounting for more than 50% in *Agkistrodon piscivorus* small intestinal samples^28^, and *Bacteroidetes* was the predominant bacterium in large intestinal samples. In this study, bacterial composition patterns of small and large intestines at the phylum level were very similar (Fig. 3), the similarity coefficients of these patterns between small and large intestines for *Deinagkistrodon*, *Naja Ptyas* and *Ptyas mucosa* were 0.98, 0.96 and 0.78, respectively. The bacterial communities were not clustered together according to gender, weight, and length (Fig. 2). We know from Supplementary Table 1, that snakes D1, D2, D3 and D8 were female *Deinagkistrodon*, and snakes D4, D5, D6 and D7 were male *Deinagkistrodon*. PCoA analyses did not separate these samples according to different sex (Fig. 2). Similar results were also found for different weights and lengths (Fig. 2). In addition, the wild snake didn’t have an obvious different bacterial community when compared to bred snakes (Fig. 2). However, *Deinagkistrodon* and *Naja* groups were clustered together with those of the *Ptyas mucosa* group being more disperse. The character and strength of venom toxicity may be one of the major factors that affected their intestinal bacterial community. Both snakes of *Deinagkistrodon* and *Naja* can secret venom (https://en.wikipedia.org/wiki/), thus the venom may have the ability to affect the growth of intestinal bacteria. However, the snake of *Ptyas mucosa* didn’t have the ability to secret venom, so there was no venom to restrict their intestinal bacterial patterns. Of course, our hypothesis requires further investigation.

Despite the fact that core microbiomes have been identified for human and mouse gut samples^31–33^, snake core gut microbes are still missing due to the small number of samples from previous snake gut microbiota studies. In this study, we sequenced 50 gut samples, and found that the core microbes existed in these snake samples, and that different breeds had different core microbiomes. As shown in Table 2, 27, 24, and 16 genera were identified as core microbes for *Deinagkistrodon*, *Naja*, and *Ptyas mucosa*, respectively. Moreover, some bacterial genera were found existing in all detected snakes (Fig. 5). In consideration that most genera listed in Table 2, like *Acinetobacter*^20^, *Aeromonas*^21^, *Citrobacter*^22^, *Morganella*^23^, *Proteus*^24^, *Providencia*^25^, *Pseudomonas*^26^, and *Salmonella*^27^ were human and animal pathogens or opportunistic pathogens, they should be defined as one of the focuses of disease prevention and control. Since *Ptyas mucosa* and *Naja* species (mostly *Naja naja*) frequent human dwellings, their feces may contaminate human and animals, and become a bacterial infection source. A total of 17 bacterial genera identified from intestines of study snakes (Table 2) were found to be pathogens or opportunistic pathogens to animals and humans (https://en.wikipedia.org/wiki/Main_Page). Therefore, this study provides an opportunity for further investigations on how snakes contribute to zoonosis in their distribution ranges.

However, some significant differences in some bacteria were identified among different breeds. More interestingly, we found that some bacteria could be related to the venom secretion. As illustrated in Fig. 6, *Hafnia_Obesumbacterium*, *Providencia*, and *Ureaplasma* were significantly higher in non-venom secretion snake *Ptyas mucosa* than the other two venom secretion snakes *Deinagkistrodon* and *Naja*. The other 7 genera, *Achromobacter*, *Cetobacterium*, *Clostridium innocuum* group, *Fusobacterium*, *Lachnoclostridium*, *Parabacteroides*, and *Romboutsia* were only detected in the venom secretion snakes *Deinagkistrodon* and *Naja*. Certainly, the function of these bacteria in venom secretion needs to be further studied. In addition, the study of the varied buccal cavity bacterial community of snakes is needed. Some pathogenic bacteria has been cultured from the venom and oropharynx of snakes, and they have been implicated in secondary infections of snake bite wounds^34,35^.

In summary, the bacterial community of 50 snake gut samples was examined using 16S rDNA sequencing analysis, and 29 bacterial phyla, 545 bacterial genera, and 1,725 OTUs were identified in these samples. Among them, 27, 24, and 16 genera were identified as core microbes for *Deinagkistrodon*, *Naja*, and *Ptyas mucosa*, respectively. In addition, the relative abundance of 3 genera (*Providencia*, *Hafnia_Obesumbacterium*, and *Ureaplasma*) were found to be significantly higher in non-venom secretion snakes, and 7 genera (*Achromobacter*, *Cetobacterium*, *Clostridium innocuum* group, *Fusobacterium*, *Lachnoclostridium*, *Parabacteroides*, *Romboutsia*) were only detected in venom secretion snakes. There is a need for additional sophisticated study to understand whether these bacteria have targets to improve venom secretion in those snakes.

## Supplementary information

Supplementary materials

## References

[1] Makki K, Deehan EC, Walter J, Backhed F (2018). The Impact of Dietary Fiber on Gut Microbiota in Host Health and Disease. Cell Host Microbe..

[2] Jandhyala SM (2015). Role of the normal gut microbiota. World J Gastroenterol..

[3] Xu WT, Nie YZ, Yang Z, Lu NH (2016). The crosstalk between gut microbiota and obesity and related metabolic disorders. Future Microbiol..

[4] Sun HL, Li CQ (2004). The Pharmacological Action and Application of Snake’s Gallbladde. r. Northwest Pharmaceutical Journal..

[5] Hao WX (1984). Effect of an antithrombotic enzyme of *Agkistrodon halys* venom in the treatment of cerebral thrombosis–report of 322 cases. Zhonghua Shen Jing Jing Shen Ke Za Zhi..

[6] Ding B (2015). Antiplatelet Aggregation and Antithrombosis Efficiency of Peptides in the Snake Venom of *Deinagkistrodon acutus*: Isolation, Identification, and Evaluation. Evid Based Complement Alternat Med..

[7] Yu MY (2017). Ozagrel sodium combined with snake anti-thrombosis enzyme therapy for cerebral hemorrhage clinical analysis. Chinese Medicine Guide..

[8] Wang, S. Z. & Qin, Z. H. Anti-Inflammatory and Immune Regulatory Actions of *Naja naja* atra Venom. *Toxins (Basel)*. **10** (2018). 10.3390/toxins10030100 PMC5869388 29495566

[9] Osipov A, Utkin Y (2012). Effects of snake venom polypeptides on central nervous system. Cent Nerv Syst Agents Med Chem..

[10] Uzair B (2018). Snake venom as an effective tool against colorectal cancer. Protein Pept Lett..

[11] Tang SD (2007). Analysis about the process and function of yongzhou *Elapidae* healthful liquor. Food Research and Development..

[12] Bin DM, Zhou JT, Zhong FS, Liu J (2006). Study on the Formula Design and Technology of Yongzhou Different snake wine. Food and Machinery..

[13] De Angelis, M. *et al*. The food-gut human axis: the effects of diet on gut microbiota and metabolome. *Curr Med Chem* (2017). 10.2174/0929867324666170428103848 28462705

[14] Koppel, N., Maini Rekdal, V. & Balskus, E. P. Chemical transformation of xenobiotics by the human gut microbiota. *Science*. **356** (2017). 10.1126/science.aag2770 PMC5534341 28642381

[15] Dennis KL (2013). Adenomatous polyps are driven by microbe-instigated focal inflammation and are controlled by IL-10-producing T cells. Cancer Res..

[16] Schloss PD (2009). Introducing mothur: open-source, platform-independent, community-supported software for describing and comparing microbial communities. Appl Environ Microbiol..

[17] Cole JR (2009). The Ribosomal Database Project: improved alignments and new tools for rRNA analysis. Nucleic Acids Res..

[18] Wang Q, Garrity GM, Tiedje JM, Cole JR (2007). Naive Bayesian classifier for rapid assignment of rRNA sequences into the new bacterial taxonomy. Appl Environ Microbiol..

[19] Segata N (2011). Metagenomic biomarker discovery and explanation. Genome Biol..

[20] Amorim AM, Nascimento JD (2017). *Acinetobacter*: an underrated foodborne pathogen?. J Infect Dev Ctries..

[21] Batra P, Mathur P, Misra MC (2016). *Aeromonas spp*.: An Emerging Nosocomial Pathogen. J Lab Physicians..

[22] Collins JW (2014). *Citrobacter rodentium*: infection, inflammation and the microbiota. Nat Rev Microbiol..

[23] Liu H, Zhu J, Hu Q, Rao X (2016). *Morganella morganii*, a non-negligent opportunistic pathogen. Int J Infect Dis..

[24] Schaffer, J. N. & Pearson, M. M. *Proteus mirabilis* and Urinary Tract Infections. *Microbiol Spectr*. **3** (2015). 10.1128/microbiolspec.UTI-0017-2013 PMC4638163 26542036

[25] Sagar S, Narasimhaswamy N, D’Souza J (2017). Providencia Rettgeri: An Emerging Nosocomial Uropathogen in an Indwelling Urinary Catheterised Patient. J Clin Diagn Res..

[26] Juan C, Pena C, Oliver A (2017). Host and Pathogen Biomarkers for Severe *Pseudomonas aeruginosa* Infections. J Infect Dis..

[27] Crowley SM, Knodler LA, Vallance BA (2016). *Salmonella* and the Inflammasome: Battle for Intracellular Dominance. Curr Top Microbiol Immunol..

[28] Leser TD (2002). Culture-independent analysis of gut bacteria: the pig gastrointestinal tract microbiota revisited. Appl Environ Microbiol..

[29] McLaughlin RW, Cochran PA, Dowd SE (2015). Metagenomic analysis of the gut microbiota of the *Timber Rattlesnake*, *Crotalus horridus*. Mol Biol Rep..

[30] Costello EK, Gordon JI, Secor SM, Knight R (2010). Postprandial remodeling of the gut microbiota in *Burmese pythons*. ISME J..

[31] Barzegari A, Saeedi N, Saei AA (2014). Shrinkage of the human core microbiome and a proposal for launching microbiome biobanks. Future Microbiol..

[32] Li J (2014). An integrated catalog of reference genes in the human gut microbiome. Nat Biotechnol..

[33] Xiao L (2015). A catalog of the mouse gut metagenome. Nat Biotechnol..

[34] Suankratay C, Wilde H, Nunthapisud P, Khantipong M (2002). Tetanus after white-lipped green pit viper (*Trimeresurus albolabris*) bite. Wilderness Environ Med..

[35] Theakston RD (1990). Bacteriological studies of the venom and mouth cavities of wild Malayan pit vipers (*Calloselasma rhodostoma*) in southern Thailand. Trans R Soc Trop Med Hyg..

