# Supplementary material for: Comparative analysis of intestinal bacteria among venom secretion and non-secrection snakes
Source: Sci Rep. 2019 Apr 19;9:6335. doi: 10.1038/s41598-019-42787-6 (PMC6474859; doi:10.1038/s41598-019-42787-6)
Supplement: Supplementary file 1 — Supplementary materials [file 41598_2019_42787_MOESM1_ESM.pdf]

Supplementary materials

Comparative analysis of intestinal bacteria among venom secretion and non-secretion snakes

Zuodong Qin[1, 2], Siqi Wang[1], Dezhi Guo[2], Jialiang Zhu[1], Huahai Chen[1], Le Bai[2], Xiaofang Luo[1], Yeshi Yin[1, *]

[1]Key Laboratory of Comprehensive Utilization of Advantage Plants Resources in Hunan South, College of Chemistry and Bioengineering, Hunan University of Science and Engineering, Yongzhou, Hunan, China

[2]Yongzhou City Strange Snake Science and Technology Industrial *Co., Ltd.*Yongzhou, Hunan, 425000, China

* Correspondence: yinyeshi@126.com

Supplementary Table 1. Basic information regarding the snakes used in this study.

Breed	No.	Rearing pattern	Gender	Weight (Kg)	Length (metre)	Sample collection time
Deinagkistrodon	D1	Wild	Female	0.75	ND	2-Nov-17
Deinagkistrodon	D2	Breed	Female	0.6	ND	2-Nov-17
Deinagkistrodon	D3	Breed	Female	0.65	ND	2-Nov-17
Deinagkistrodon	D4	Breed	Male	0.25	ND	2-Nov-17
Deinagkistrodon	D5	Breed	Male	0.4	ND	10-Nov-17
Deinagkistrodon	D6	Breed	Male	0.4	ND	10-Nov-17
Deinagkistrodon	D7	Breed	Male	0.4	ND	10-Nov-17
Deinagkistrodon	D8	Breed	Female	0.45	ND	10-Nov-17
Naja	N1	Wild	Male	0.5	1.05	30-Sep-17
Naja	N2	Breed	Female	0.5	0.95	30-Sep-17
Naja	N3	Wild	Male	0.5	1.1	30-Sep-17
Naja	N4	Wild	Male	0.5	1.05	30-Sep-17
Naja	N5	Breed	Male	0.6	ND	2-Nov-17
Naja	N6	Breed	Male	0.6	ND	2-Nov-17
Naja	N7	Breed	Female	0.55	ND	2-Nov-17
Naja	N8	Breed	Male	0.45	ND	10-Nov-17
Naja	N9	Breed	Male	0.55	ND	10-Nov-17
Naja	N10	Breed	Male	1.4	ND	10-Nov-17
Naja	N11	Breed	Male	1.3	ND	10-Nov-17
Naja	N12	Breed	Male	ND	ND	16-Nov-17
Naja	N13	Breed	Male	ND	ND	16-Nov-17
Naja	N14	Breed	Male	ND	ND	16-Nov-17
Ptyas mucosa	P1	Breed	Male	1.25	2.1	30-Sep-17
Ptyas mucosa	P2	Breed	Male	1.2	ND	10-Nov-17
Ptyas mucosa	P3	Breed	Male	1.2	ND	10-Nov-17
Ptyas mucosa	P4	Breed	Female	0.9	ND	10-Nov-17
Ptyas mucosa	P5	Breed	Male	ND	ND	16-Nov-17
Ptyas mucosa	P6	Breed	Male	ND	ND	16-Nov-17
Ptyas mucosa	P7	Breed	Female	ND	ND	16-Nov-17

D, N, and P represents *Deinagkistrodon*, *Naja*, and *Ptyas mucosa*, respectively. ND represents not detected.

Supplementary Table 2. Basic statistical results for high-throughput 16S rRNA gene sequencing.

Sample ID	Reads	OTU (0.97)	Coverage	Ace	Chao	Shannon	Simpson
D1_I	102324	359	99.95%	382.52	406.05	2.47	0.23
D2_I	95640	345	99.97%	356.66	360.12	2.19	0.25
D3_I	95438	352	99.92%	381.02	372.07	2.54	0.14
D3_L	145406	198	99.96%	306.01	321.75	2.44	0.14
D4_I	83510	256	99.92%	376.01	343.65	3.65	0.05
D4_L	75579	270	99.97%	281.70	285.81	2.67	0.17
D5_L	70757	211	99.94%	285.85	286.25	3.38	0.08
D6_I	73958	104	99.95%	255.95	201.50	2.18	0.17
D6_L	75368	294	99.90%	363.89	373.64	3.03	0.10
D7_I	147420	495	99.93%	570.46	581.67	2.06	0.26
D7_L	120619	366	99.95%	415.68	416.83	3.93	0.04
D8_I	140727	243	99.95%	312.66	318.68	2.23	0.21
D8_L	74611	386	99.83%	498.60	500.77	3.72	0.05
N1_I	63319	150	99.91%	310.06	245.06	2.00	0.22
N1_L	96753	274	99.92%	412.41	363.52	3.01	0.12
N2_I	86450	517	99.83%	626.55	636.28	3.95	0.04
N2_L	99001	289	99.93%	362.72	354.17	3.14	0.09
N3_I	81141	329	99.88%	416.86	404.48	3.22	0.07
N3_L	94750	241	99.96%	267.62	280.18	2.05	0.27
N4_I	116403	372	99.97%	386.74	418.50	3.29	0.09
N4_L	142531	215	99.97%	257.68	253.33	2.51	0.16
N5_I	102463	298	99.91%	474.23	402.65	3.07	0.07
N5_L	122811	289	99.94%	365.00	369.16	2.37	0.19
N6_I	114964	435	99.93%	495.37	491.84	3.09	0.11
N7_L	120256	180	99.95%	313.84	270.05	2.77	0.09
N8_I	98403	181	99.93%	359.84	277.13	2.57	0.11
N8_L	116670	300	99.95%	347.65	334.88	3.05	0.07
N9_I	113063	458	99.92%	512.36	512.60	3.09	0.13
N9_L	97692	513	99.95%	540.12	550.12	4.35	0.03
N10_I	17370	375	99.59%	427.31	420.18	4.18	0.04
N10_L	97971	213	99.95%	334.51	278.62	3.16	0.07
N11_I	86405	330	99.89%	370.55	370.23	2.64	0.16
N11_L	106417	431	99.91%	511.92	511.72	3.38	0.09
N12_I	79430	513	99.82%	611.88	605.38	3.47	0.07
N12_L	92712	280	99.93%	397.39	339.09	3.62	0.04
N13_I	94335	170	99.95%	288.77	243.50	3.05	0.07
N13_L	87453	456	99.88%	567.30	544.06	4.09	0.03
N14_I	69778	238	99.86%	387.08	346.94	2.50	0.13

N14_L	62933	258	99.88%	404.24	350.04	3.49	0.06
P1_I	116923	265	99.97%	287.72	298.48	2.52	0.18
P1_L	100767	108	99.97%	237.25	156.00	2.88	0.09
P2_L	102193	131	99.96%	228.85	177.87	2.57	0.14
P3_I	86101	202	99.93%	320.85	277.14	2.83	0.10
P3_L	75856	91	99.97%	113.40	133.00	2.59	0.13
P5_I	101218	312	99.96%	334.33	341.64	2.06	0.34
P5_L	86702	200	99.93%	278.29	254.03	2.97	0.07
P6_I	65208	335	99.92%	370.04	368.28	2.91	0.11
P6_L	69632	228	99.93%	277.83	269.44	2.51	0.16
P7_I	64615	122	99.94%	205.15	189.36	1.98	0.19
P7_L	101834	289	99.91%	387.01	391.69	2.60	0.11

D, N, and P represents *Deinagkistrodon*, *Naja*, and *Ptyas mucosa*, respectively. I and L represent ileum and large intestinal samples, respectively.

Supplementary Figure 1. Basic analysis of sequenced data.

A, rarefaction curves of sequenced samples. B, the Shannon-Wiener curves of sequenced samples. C, Specaccum analysis of sequenced samples. D, Rank-abundance distribution curve of sequenced samples.

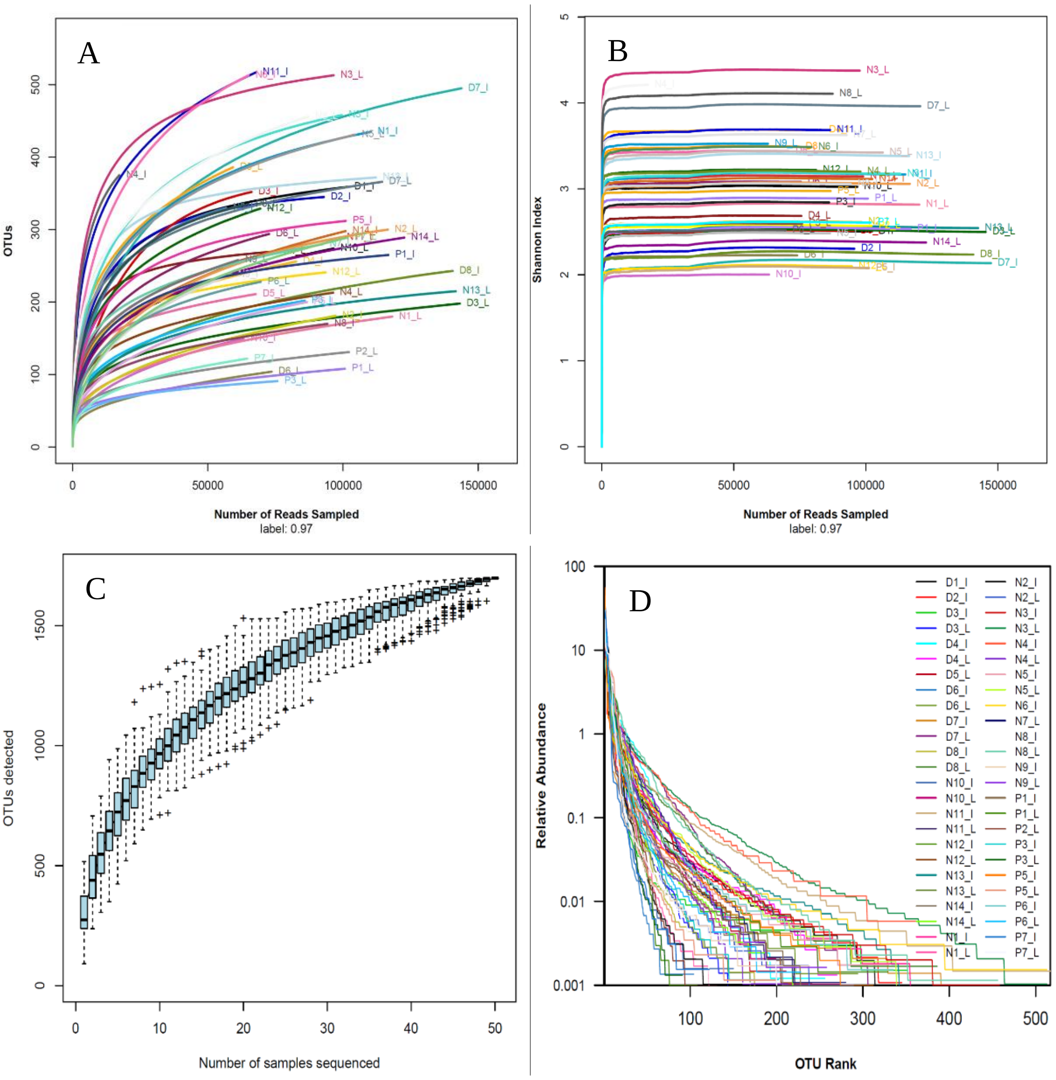

Supplementary Figure 2. Distribution of the core bacterial phyla.

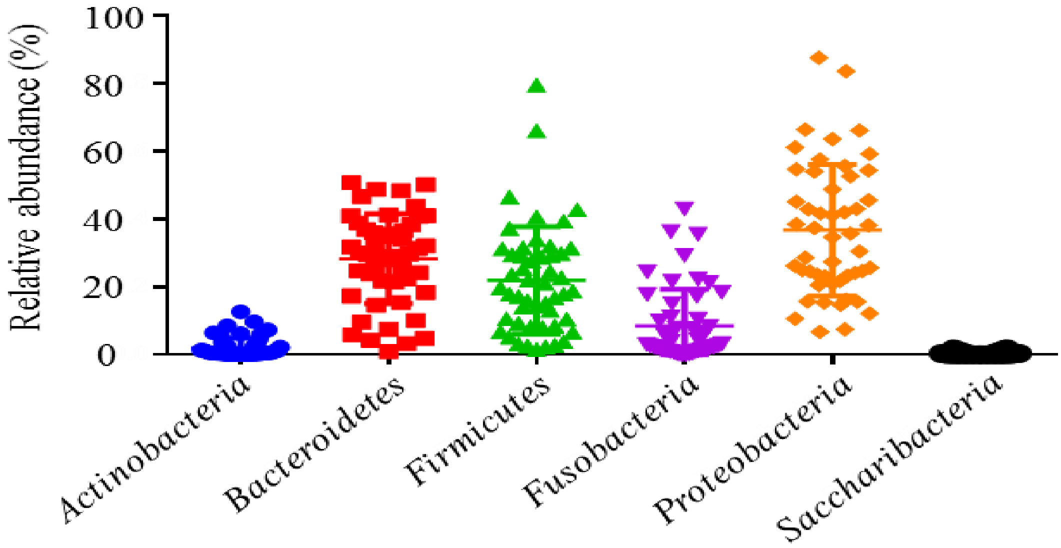

Supplementary Figure 3. Significantly different bacterial phyla between snake breeds.

A, different bacterial phyla between *Deinagkistrodon* to *Naja* samples; B, different bacterial phyla between *Deinagkistrodon* to *Ptyas mucosa* samples; C, different bacterial phyla between *Naja* to *Ptyas mucosa* samples.

A

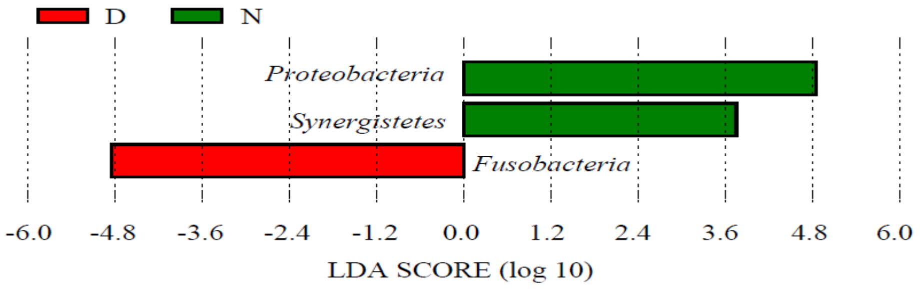

B

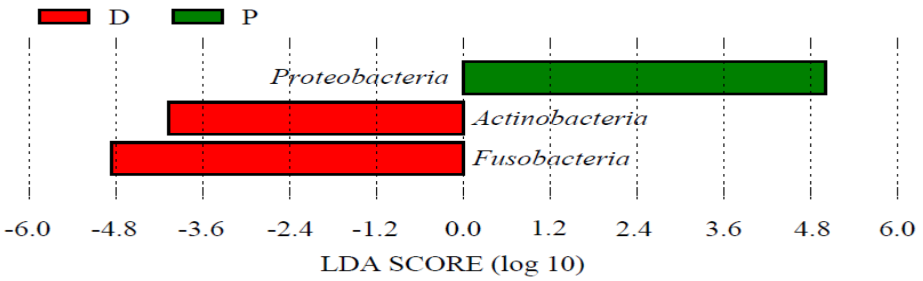

C

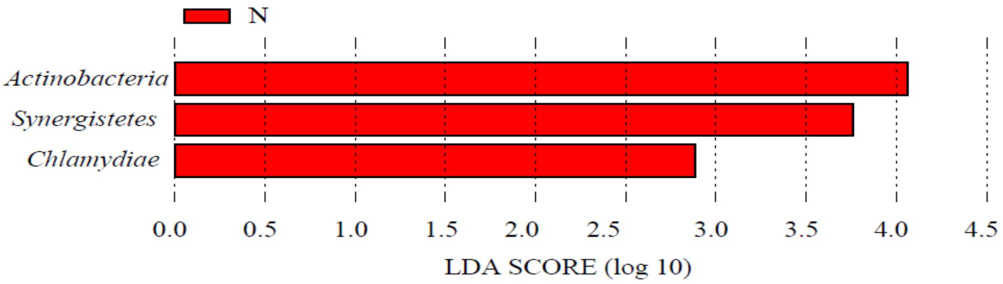

Supplementary Figure 4. Distribution of the core bacterial genus.

A, core bacterial genus in *Deinagkistrodon* samples; B, core bacterial genus in *Naja* samples; C, core bacterial genus in *Ptyas mucosa* samples.

A

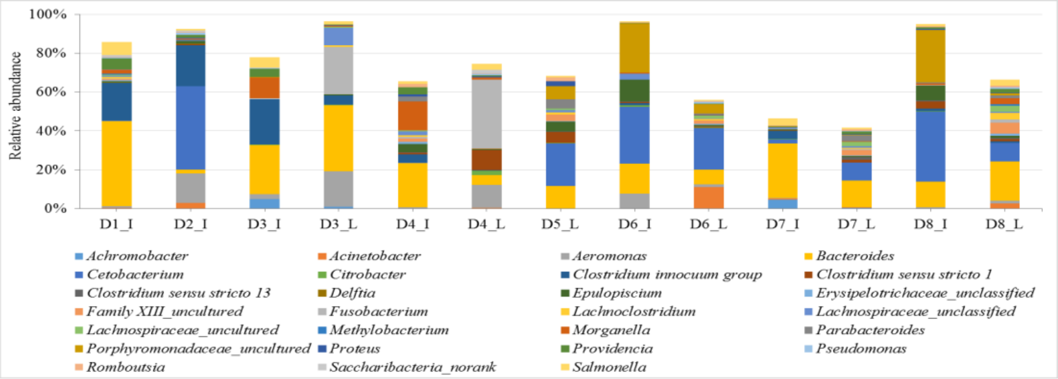

B

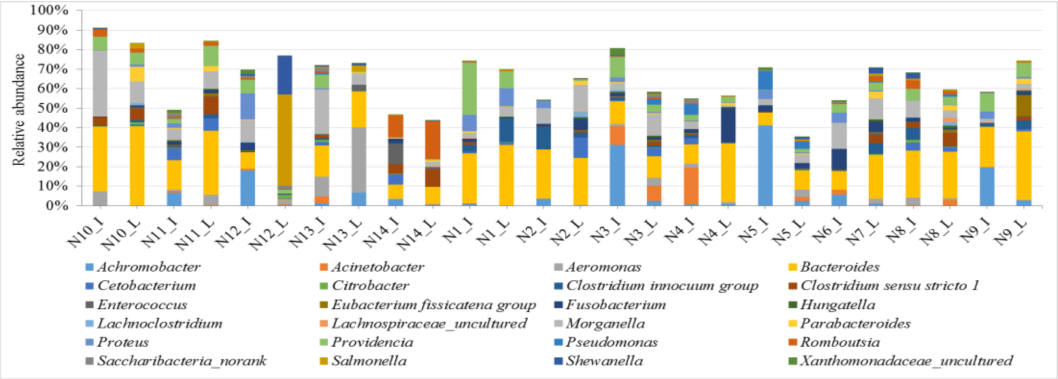

C

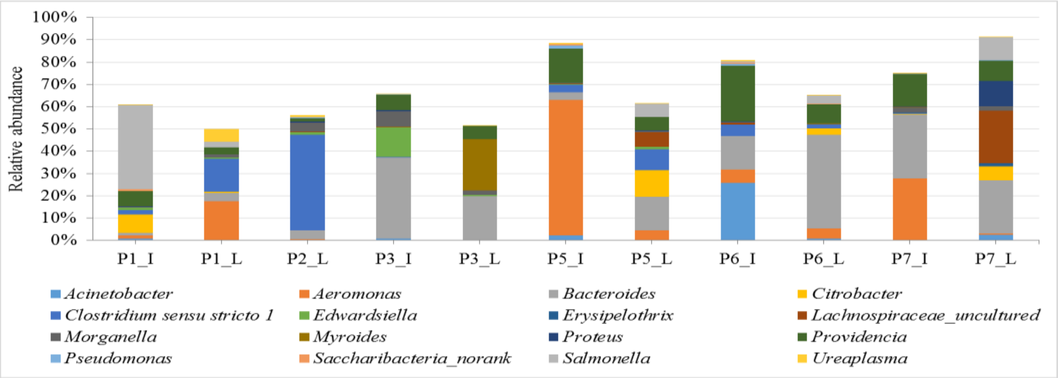

Supplementary Figure 5. Significantly different bacterial genus between snake breeds.

A, different bacterial genus between *Deinagkistrodon* to *Naja* samples; B, different bacterial genus between *Deinagkistrodon* to *Ptyas mucosa* samples; C, different bacterial genus between *Naja* to *Ptyas mucosa* samples.

A

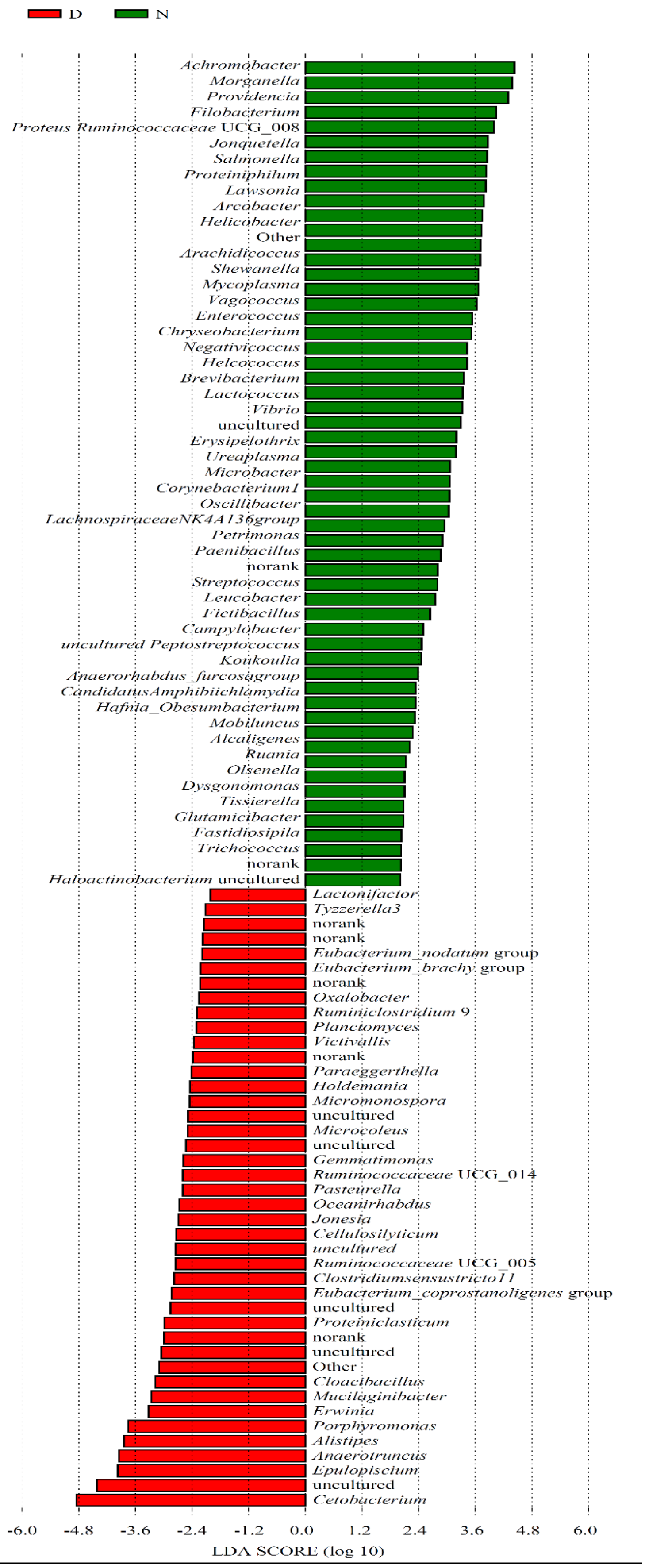

D
N
Achromobacter
Morganella
Providencia
Filobacterium
Proteus Ruminococcaceae UCG_008
Jonquetella
Salmonella
Proteiniphilum
Lawsonia
Arcobacter
Helicobacter
Other
Arachidicoccus
Shewanella
Mycoplasma
Vagococcus
Enterococcus
Chryseobacterium
Negativicoccus
Helcococcus
Brevibacterium
Lactococcus
Vibrio
uncultured
Erysipelothrix
Ureaplasma
Microbacter
Corynebacterium1
Oscillibacter
LachnospiraceaeNK4A136group
Petrimonas
Paenibacillus
norank
Streptococcus
Leucobacter
Fictibacillus
Campylobacter
uncultured Peptostreptococcus
Koukoulia
Anaerorhabdus_furcosagroup
CandidatusAmphibiichlamydia
Hafnia_Obesumbacterium
Mobiluncus
Alcaligenes
Ruania
Olsenella
Dysgonomonas
Tissierella
Glutamicibacter
Fastidiosipila
Trichococcus
norank
Haloactinobacterium uncultured
Lactonifactor
Tyzzerella3
norank
norank
Eubacterium_nodatum group
Eubacterium_brachy group
norank
Oxalobacter
Ruminiclostridium 9
Planctomyces
Victivallis
norank
Paraeggerthella
Holdemania
Micromonospora
uncultured
Microcoleus
uncultured
Gemmatimonas
Ruminococcaceae UCG_014
Pasteurella
Oceanirhabdus
Jonesia
Cellulosilyticum
uncultured
Ruminococcaceae UCG_005
Clostridiumsensustricto11
Eubacterium_coprostanoligenes group
uncultured
Proteiniclasticum
norank
uncultured
Other
Cloacibacillus
Mucilaginibacter
Erwinia
Porphyromonas
Alistipes
Anaerotruncus
Epulopiscium
uncultured
Cetobacterium
-6.0
-4.8
-3.6
-2.4
-1.2
0.0
1.2
2.4
3.6
4.8
6.0
LDA SCORE (log 10)

B

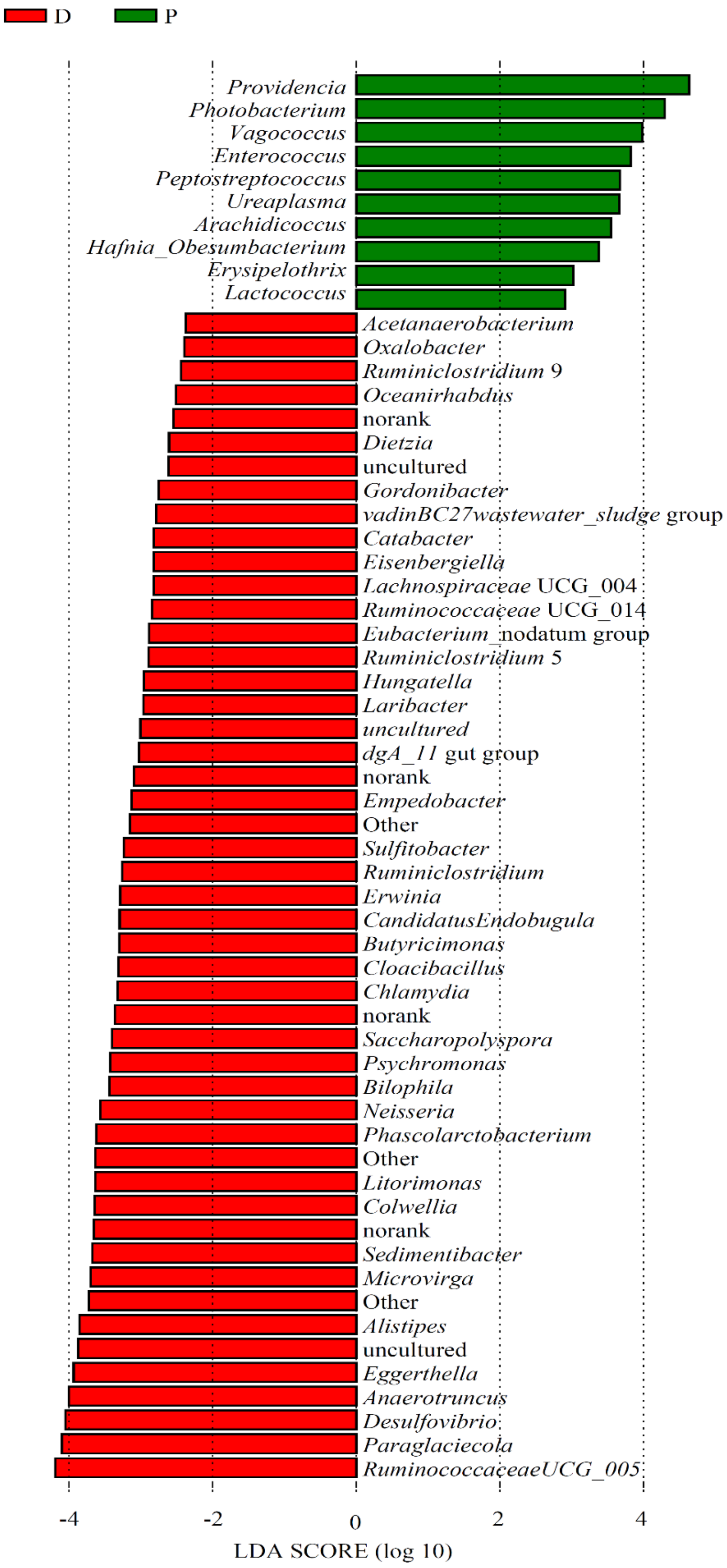

D
P
Providencia
Photobacterium
Vagococcus
Enterococcus
Peptostreptococcus
Ureaplasma
Arachidicoccus
Hafnia_Obesumbacterium
Erysipelothrix
Lactococcus
Acetanaerobacterium
Oxalobacter
Ruminiclostridium 9
Oceanirhabdus
norank
Dietzia
uncultured
Gordonibacter
vadinBC27wastewater_sludge group
Catabacter
Eisenbergiella
Lachnospiraceae UCG_004
Ruminococcaceae UCG_014
Eubacterium_nodatum group
Ruminiclostridium 5
Hungatella
Laribacter
uncultured
dgA_11 gut group
norank
Empedobacter
Other
Sulfitobacter
Ruminiclostridium
Erwinia
CandidatusEndobugula
Butyricimonas
Cloacibacillus
Chlamydia
norank
Saccharopolyspora
Psychromonas
Bilophila
Neisseria
Phascolarctobacterium
Other
Litorimonas
Colwellia
norank
Sedimentibacter
Microvirga
Other
Alistipes
uncultured
Eggerthella
Anaerotruncus
Desulfovibrio
Paraglaciecola
RuminococcaceaeUCG_005
-4
-2
0
2
4
LDA SCORE (log 10)

C

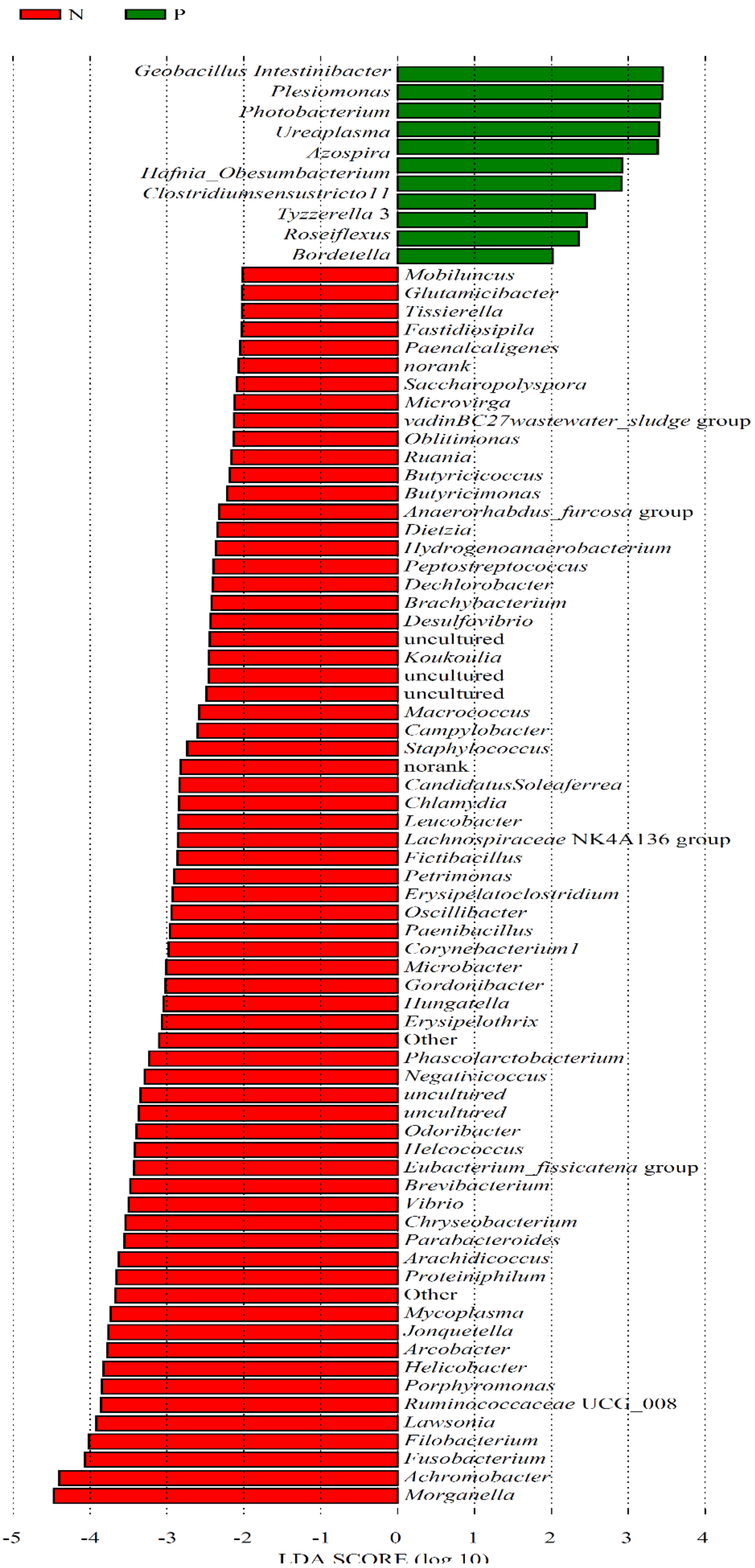

N
P
Geobacillus Intestinibacter
Plesiomonas
Photobacterium
Ureaplasma
Azospira
Hafnia_Obesumbacterium
Clostridiumsensustricto11
Tyzzerella 3
Roseiflexus
Bordetella
Mobiluncus
Glutamicibacter
Tissierella
Fastidiosipila
Paenalcaligenes
norank
Saccharopolyspora
Microvirga
vadinBC27wastewater_sludge group
Oblitimonas
Ruania
Butyricicoccus
Butyricimonas
Anaerorhabdus_furcosa group
Dietzia
Hydrogenoanaerobacterium
Peptostreptococcus
Dechlorobacter
Brachybacterium
Desulfovibrio
uncultured
Koukoulia
uncultured
uncultured
Macrococcus
Campylobacter
Staphylococcus
norank
CandidatusSoleaferrea
Chlamydia
Leucobacter
Lachnospiraceae NK4A136 group
Fictibacillus
Petrimonas
Erysipelatoclostridium
Oscillibacter
Paenibacillus
Corynebacterium1
Microbacter
Gordonibacter
Hungatella
Erysipelothrix
Other
Phascolarctobacterium
Negativicoccus
uncultured
uncultured
Odoribacter
Helcococcus
Eubacterium_fissicatena group
Brevibacterium
Vibrio
Chryseobacterium
Parabacteroides
Arachidicoccus
Proteiniphilum
Other
Mycoplasma
Jonquetella
Arcobacter
Helicobacter
Porphyromonas
Ruminococcaceae UCG_008
Lawsonia
Filobacterium
Fusobacterium
Achromobacter
Morganella
-5
-4
-3
-2
-1
0
1
2
3
4
LDA SCORE (log 10)
